# Supplementary material for: Chromothripsis during telomere crisis is independent of NHEJ, and consistent with a replicative origin
Source: Genome Res. 2019 May;29(5):737–49. doi: 10.1101/gr.240705.118 (PMC6499312; doi:10.1101/gr.240705.118)
Supplement: Supplemental Material [file supp_gr.240705.118_Supplemental_file_1.zip › contigs/annotated_contigs/DB51/contig.4.DB51_length_1091_mean_cov_6.85976168653.docx]

**DB51_length_1091_mean_cov_6.85976168653**

```
TGAGTACAGGTGGGAAATTATGCCCACATGCAAATGTCCCCAGGGGCGGGATAAGGTCACAGCACTAGGAACCACTAGGCAAGAAGATC
 >chr19:12524722-12525201 - E=5e-270
GCTCTGTGAGAGTCTGGGTCTGGAACCTGTTTTTCTGCCTCCACTCAGCACCAAGAGATACCTTCTTCTGTCACTGAGTTCCCCAGGGG

GCGGGACATGGTACACTACTCAATCAGGGACGTGGGGTGGGGTCGTGAGAACTGTGAATAAGGGAGCTGCCGCGAGGAGGCTGTGAGGT

TCCAAGAGCCCAGGGCATCTTCTCCACTCCTTGCGCTTGGTTCCCAGCTCTGTTGCTAAGAGACGCCCTGAAAGTTCTGTGGCAGCCTC

TGGCACACTGGGACCCGCACAGGCCGCAGGAGCCGTAGAGAAGACCCCGGGACACCGTGGAAGCCAGGAAATGGTGCGTGTGCACGGCC

TAGGGGTCCGGAGACGGGAGGAGGGGTTGGTT|GG|GACATGGTGACAGAAAGATGGTTACGTAAGGGAGATATCTGGATCAACCACAT
                                 >chr19:12522496-12522881 - E=2e-204 p=4e-02
TGAGGAAGGAGGAGGAGGTAGAGAACAAGAAAGTGTATCTGAGATTACAAACCCATTTTCTGCTTGGAGAGTGTTCAAAGCAAATTCAA

AATGGAAGCTGGGCTGGGCACAGTGGCTCACACCTGTAATCTTAGCACTTTGGGAGGCTGAGGCTGGCAGATCACTTGAGACCAGGAGT

TCGATACCAGCCTGACCAACGTGGTAAAACCCTGTATCTACTAAAAATACAAAAATTAGCTGGGTGTGGTGGTGCACATCTGTAGTCCC

AGCTGCTCAGGAGTCTGAGGCAGGAAAATTGCTTGAACCTGGGAGGTGGAGGTTGCAGTGAGC|CAAGGTTGCA|CCACTGGACTCCAG
                                                                          >chr3:1951549
CCTGGGTGACAGAGCAAGATCCTGTC|TCAAAAAACAAAA|CTAAGAACATACTATACATATCATGAAACTGATGTTGTCACTAAGCAA
68-195155021 - E=7e-17 p=0e+00 >chr4:67149444-67149623 - E=1e-95
AATATTGTGAAATATTCTCCAGACAAGAAATTATTGTCTCCAAAGCCATTGGTCTATATCTAAAATAGAATACTACTTACATATATATG

TATAGTATTCTAGATGGAGTAAACTAGAA
```
